# Early prediction model for in-hospital all-cause death in patients with acute ST-elevation myocardial infarction after primary coronary artery stenting

**DOI:** 10.3389/fcvm.2026.1748053

**Published:** 2026-05-19

**Authors:** Ying Zhou, Fei Dong, Yu-Fei Zhao, Li-Jun Xiao, Yun-Qiang Zhang, Hai-Qing Liang, Mu Guo, Rui Jing, Yu Song

**Affiliations:** 1 Department of Heart Failure, TEDA International Cardiovascular Hospital, Tianjin University, Tianjin, China; 2 Department of CCU, TEDA International Cardiovascular Hospital, Tianjin University, Tianjin, China; 3 Department of Cardiology, Shanghai Fourth People’s Hospital, Shanghai, China

**Keywords:** ST-segment elevation myocardial infarction, primary coronary artery stenting, in-Hospital all-cause death, early prediction model, bootstrap internal validation

## Abstract

**Background:**

The in-hospital mortality for acute ST-segment elevation myocardial infarction (STEMI) patients remains high. This study aims to establish an early prediction model for in-hospital all-cause death in patients with STEMI after primary coronary artery stenting.

**Methods:**

This retrospective study analyzed 3,916 STEMI patients undergoing primary coronary stenting within 24 h of symptom onset at TEDA International Cardiovascular Hospital (2014–2022). We collected demographic, clinical, and procedural data, along with 48-h laboratory results (including echocardiography and Holter monitoring). The primary outcome was in-hospital all-cause mortality. Eighty clinical parameters were compared between survivors and non-survivors to identify risk factors and develop an early prediction model.

**Results:**

In a cohort of 3,916 patients, 54 experienced in-hospital all-cause death. Comparison of 80 clinical variables between groups, followed by univariate logistic regression and least absolute shrinkage and selection operator (LASSO) regression, identified nine risk factors. Multicollinearity analysis confirmed no significant interactions. Multivariate logistic regression revealed six independent predictors: B-type natriuretic peptide (BNP) (per 200 pg/mL), creatine kinase-MB isoenzyme (CK-MB) (per 100 ng/mL), blood urea nitrogen (BUN) (per 1 mmol/L), lactate (LAC) (per 1 mmol/L), Holter mean heart rate (MHR) (per 10 bpm), and Holter total atrial beats (TAB) (per 1,000 times). Receiver operating characteristic (ROC) curve, calibration slope, decision curve analysis (DCA) demonstrated superior net benefit of the combined model over individual predictors. Twenty-five cross-validations confirmed excellent performance of this prediction model, with mean AUC of 0.911 and good stability. Bootstrap internal validation confirmed the good stability (optimism-corrected AUC = 0.873, optimism = 0.019) and calibration (calibration slope = 1.061) of the model, indicating that despite a borderline events per variable (EPV) of 9, the model can reliably predict in-hospital mortality risk in STEMI patients.

**Conclusion:**

The combination of BNP, CK-MB, BUN, LAC, Holter MHR, and Holter TAB can effectively predict in-hospital all-cause death in STEMI patients undergoing primary coronary artery stenting, offering potential clinical utility.

## Introduction

Cardiovascular diseases (CVDs) continue to exhibit rising prevalence and mortality rates, standing as a leading cause of death and disability worldwide. Acute ST-elevation myocardial infarction (STEMI), one of the most dangerous manifestations of coronary artery disease, has become a major health concern. Despite significant advancements in treatment, particularly the widespread adoption of primary percutaneous coronary intervention (PPCI), the in-hospital mortality for STEMI patients remains as high as 8.6% (1).

Acute myocardial infarction (AMI) causes impaired energy metabolism, inflammatory responses, oxidative stress, ischemia—reperfusion injury, myocardial hypertrophy, and fibrosis in cardiomyocytes, leading to abnormal myocardial remodeling and electrical instability (2). Delayed hospital arrival, patient refusal of PPCI due to financial or physical constraints, or complications such as heart failure (HF), cardiogenic shock, malignant arrhythmias, mechanical complications, reinfarction, bleeding, stroke or infection may ultimately lead to in-hospital death. Early identification of high-risk features for in-hospital mortality is therefore critical to improving STEMI outcomes.

Currently, there is a lack of early predictive models for in-hospital mortality among STEMI patients undergoing primary coronary stenting. This study aims to evaluate the predictive value of early clinical indicators for in-hospital all-cause mortality in this population.

## Methods

### Subjects

This study retrospectively analyzed 3,916 patients presenting within 24 h of symptom onset, who were diagnosed with STEMI and treated with primary coronary stenting at TEDA International Cardiovascular Hospital between October 2014 and December 2022. The STEMI diagnosis criteria followed the 2013 American College of Cardiology Foundation (ACCF)/American Heart Association (AHA) STEMI Management Guidelines and the 2017 European Society of Cardiology (ESC) STEMI Diagnosis and Treatment Guidelines (3, 4).

**Figure 1:**
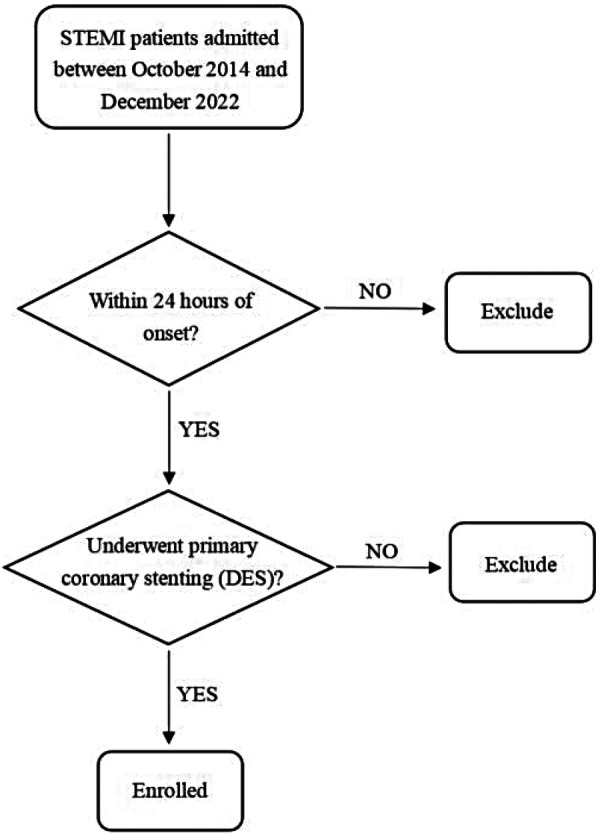
Flow diagram of patient enrollment. STEMI, ST-segment elevation myocardial infarction; DES, drug—eluting stent.

All enrolled patients received drug-eluting stents (DES), with the coronary stenting procedures performed according to the 2014 ESC/European Association for Cardio-Thoracic Surgery (EACTS) Guidelines on Myocardial Revascularization and the 2021 American College of Cardiology (ACC)/AHA/Society for Cardiovascular Angiography and Interventions (SCAI) Coronary Artery Revascularization Guidelines (5, 6). Preoperative and postoperative management adhered to standardized protocols as outlined in the guidelines (3, 4). A flowchart of patient enrollment is presented in Figure 1.

### Data collection

General information: gender, age, body mass index (BMI).

Medical history: onset time, history of hypertension, diabetes mellitus, smoking status, and alcohol consumption was collected.

Initial vital signs: temperature, heart rate (HR), respiratory rate (RR), blood pressure (BP), and peripheral oxygen saturation (SpO_2_) were recorded.

Killip classification at admission.

Intraoperative data: Door-to-wire time (D-to-W time), infarct-related artery (IRA), IRA pre-PCI thrombolysis in myocardial infarction (TIMI) flow grade, IRA post-PCI TIMI flow grade and number of stents implanted were collected.

Laboratory tests: cardiac biomarkers [high-sensitivity cardiac troponin I (cTNI), myoglobin (MYO), creatine kinase-MB isoenzyme (CK-MB)], B-type natriuretic peptide (BNP), lactate (LAC), full blood count, renal and liver function tests, electrolytes, lipid profiles, random blood glucose (RBG), C-reactive protein (CRP), thyroid function tests, bedside echocardiography, and 24-h Holter monitoring. All these tests (except Holter) were completed within 24 h of hospital admission, with cardiac biomarkers recorded at their peak within this period. The Holter was performed within 48 h. The estimated glomerular filtration rate (eGFR) was calculated using the modified simplified modification of diet in renal disease (MDRD) formula (7).

### Clinical outcomes

The primary clinical outcome was in-hospital all-cause mortality. In-hospital death included patients who died during hospitalization as well as those discharged in a terminal condition. Patients were categorized into either the in-hospital survival group or death group. All date can be obtained within 48 h of admission in STEMI patients undergoing emergency PCI, comparative analysis of clinical parameters between these two groups was performed to identify early independent predictors. Subsequently, an early prediction model (the first 48 h of hospitalization) was developed and its predictive performance was rigorously evaluated, which is aimed to identify high-risk patients who may benefit from intensified monitoring and therapeutic strategies.

### Statistical analysis

Data were collected between October 2014 and December 2022. Missing data (9.8%) were handled using multiple imputation with chained equations (MICE) in SPSS, generating 5 imputed datasets. The imputation model included a majority of variables in the dataset, including the outcome and all key predictors. Normality was assessed using the Kolmogorov–Smirnov test. Continuous data are presented as mean ± SD or median (P25, P75), and categorical data as percentages. Group comparisons were performed using t-tests, Mann–Whitney *U*, *χ*^2^, or rank-sum tests as appropriate. Given the limited number of outcome events (*n* = 54), a preliminary dimensionality reduction step was performed by retaining variables with *p* < 0.05 from univariate analysis. Variable selection was then performed using least absolute shrinkage and selection operator (LASSO) regression. After confirming no multicollinearity, the LASSO-selected variables were included in a multivariate logistic regression model to estimate odds ratios (no stepwise selection was applied), visualized via forest plot and nomogram. After confirming no multicollinearity, selected variables were included in a multivariate logistic regression model (forward-LR) to build a prediction model, visualized via forest plot and nomogram. Model performance was evaluated by ROC analysis, calibration slope, Brier score, decision curve analysis (DCA), with AUC comparisons made using DeLong's test. Internal validation was conducted using repeated stratified 5-fold cross-validation (25 repeats). To assess overfitting risk, internal validation was performed (1,000 repetitions). Optimism-corrected AUC was calculated by subtracting the average optimism from the apparent AUC. The 95% CI was derived from the bootstrap distribution. Calibration was assessed using the calibration slope.

Statistical analyses were performed using SPSS 27 (IBM Corporation, Armonk, NY, USA), R4.4 (R Foundation for Statistical Computing, Vienna, Austria; utilizing glmnet, rmda, pROC, boot, rms, ggplot2 and ggprism packages) and Python3.9 (Python Software Foundation, Wilmington, DE, USA; utilizing pandas, scikit-learn, matplotlib libraries). A *p*-value <0.05 was considered statistically significant.

## Results

### Basic data

Among the 3,916 patients [median age 61 years (IQR: 52–69); 3,000 males (76.6%) and 916 females (23.4%)], 54 experienced in-hospital mortality, with causes including mechanical complications (*n* = 21), cardiogenic shock (*n* = 14), malignant arrhythmias (*n* = 4), multiorgan failure (*n* = 13), and septic shock (*n* = 2).

Compared with survivors, non-survivors were older, more often female, and had higher rates of hemodynamic instability, intra—aortic balloon pump (IABP) and temporary pacemaker (TPM) use, and higher Killip class (≥2). They also showed significantly higher levels of multiple cardiac, inflammatory, and metabolic biomarkers, but lower BMI, blood pressures, left ventricular ejection fraction (LVEF), RBC-related indices, eGFR, albumin (ALB), and triglycerides.

No significant differences were found in medical history, smoking/alcohol use, D-to-W time, stent numbers, medication use [e.g., β-receptor blockers (β-B), intraprocedural use of glycoprotein IIb/IIIa inhibitors], or in various laboratory parameters including lipids, electrolytes, and liver function markers [except alanine minotransferase (ALT)/aspartate aminotransferase (AST)]. For detailed data, refer to Table 1.

**Table 1:** Comparison of baseline characteristics of patients.

Variable	Total (*n* = 3,916)	Survival group (*n* = 3,862)	Death group (*n* = 54)	*P* value
Age (years)	61 (52, 69)	61 (52,69)	75 (61, 80)	<0.001
Women (%)	916 (23.4)	896 (23.2)	20 (37.0)	0.017
BMI (kg/m^2^)	25.2 (23.2, 27.2)	25.2 (23.2, 27.2)	23.5 (21.0, 25.6)	<0.001
Current smoking (%)	2,178 (55.6)	2,154 (55.8)	24 (44.4)	0.096
Drinking history (%)	1,139 (29.1)	1,123 (29.1)	16 (29.6)	0.929
Medical history				
Hypertension (%)	2,217 (56.6)	2,185 (56.6)	32 (59.3)	0.693
Diabetes (%)	906 (23.1)	889 (23.0)	17 (31.5)	0.143
Stroke (%)	417 (10.6)	412 (10.7)	5 (9.3)	0.739
Atrial Fibrillation (%)	56 (1.4)	54 (1.4)	2 (3.6)	0.156
Gastrointestinal Diseases (%)	258 (6.6)	253 (6.6)	5 (9.3)	0.426
Kidney Disease (%)	66 (1.7)	64 (1.7)	2 (3.7)	0.246
Gallbladder Disease(%)	66 (1.7)	64 (1.7)	2 (3.7)	0.246
Lung Disease (%)	63 (1.6)	62 (1.6)	1 (1.9)	0.886
Thyroid Disease (%)	46 (1.2)	46 (1.2)	0 (0)	0.420
Gout (%)	34 (0.9)	33 (0.9)	1 (1.9)	0.433
Peripheral Vascular Disease (%)	43 (1.1)	42 (1.1)	1 (1.9)	0.592
Prior Myocardial Infarction (%)	222 (5.7)	219 (5.7)	3 (5.6)	0.971
PCI (%)	321 (8.2)	317 (8.2)	4 (7.4)	0.831
CABG (%)	11 (0.3)	11 (0.3)	0 (0)	0.695
Coronary Heart Disease (%)	376 (9.6)	370 (9.6)	6 (11.1)	0.705
Onset time (h)	3.0 (2.0,5.5)	3.0 (2.0,5.5)	3.0 (2.0,6.0)	0.890
Initial vital signs				
HR (bpm)	73 (62,82)	73 (62,82)	80 (68,97)	0.013
RR (times/ minute)	18 (18,19)	18 (18,19)	18 (18,22)	0.051
SBP (mmHg)	138 (120,154)	139 (121,154)	120 (97,141)	<0.001
DBP (mmHg)	83 (70,93)	83 (70,93)	70 (60,90)	<0.001
MAP (mmHg)	102 (90,113)	102 (90,113)	88 (73,107)	<0.001
SpO_2_ (%)	98 (97,99)	98 (97,99)	98 (94,99)	<0.001
Emergency hemodynamic instability (%)	282 (7.2)	264 (6.8)	18 (33.3)	<0.001
D-to-W time (min)	54 (44,66)	54 (44,66)	54 (47,65)	0.706
IRA				
LM (%)	31 (0.8)	25 (0.8)	6 (11.1)	<0.001
LAD (%)	1,806 (46.1)	1,778 (46.0)	28 (51.9)	
LCX (%)	432 (11.0)	428 (11.1)	4 (7.4)	
RCA (%)	1,644 (42.0)	1,628 (42.2)	16 (29.6)	
Intermediate branch (%)	3 (0.1)	3 (0.1)	0 (0)	
IRA pre -PCI TIMI flow grade				
Class 0 (%)	774 (19.8)	769 (19.9)	5 (9.3)	0.071
Class 1 (%)	257 (6.6)	254 (6.6)	3 (5.6)	
Class 2 (%)	341 (8.7)	339 (8.8)	2 (3.7)	
Class 3 (%)	2,544 (65.0)	2,500 (64.7)	44 (81.5)	
IRA pos -PCI TIMI flow grade				
Class ≦2 (%)	74 (1.9)	71 (1.8)	3 (5.6)	0.046
Class 3 (%)	3,842 (98.1)	3,791 (98.2)	51 (94.4)	
Number of stents implanted				
1 (%)	3,130 (79.9)	3,090 (80.0)	40 (74.1)	0.237
2 (%)	719 (18.4)	705 (18.3)	14 (25.9)	
≥3 (%)	67 (1.7)	67 (1.7)	0 (0)	
Intraoperative use				
IABP (%)	79 (2.0)	69 (1.8)	10 (18.5)	<0.001
TPM (%)	42 (1.1)	37 (1.0)	5 (9.3)	<0.001
GP IIb/IIIa inhibitors (%)	528 (13.5)	520 (13.5)	8 (14.8)	0.773
Vasoactive agents (%)	290 (7.4)	284 (7.4)	6 (11.1)	0.295
ACEI/ARB/ARNI use within 24 h of admission (%)	631 (16.1)	623 (16.1)	8 (14.8)	0.794
β-B use within 24 h of admission (%)	1,166 (29.8)	1,158 (30.0)	8 (14.8)	0.015
LVEF (%)	55 (50, 60)	55 (50,60)	46 (35, 53)	<0.001
BNP (pg/mL)	66 (23, 168)	65 (23,166)	174 (55, 642)	<0.001
cTnI (pg/mL)	26.8 (13.5, 56.2)	26.6 (13.3, 55.1)	71.9 (27.9, 81.0)	<0.001
MYO (ng/mL)	95 (46, 216)	93 (46, 206)	747 (345, 1,311)	<0.001
CK-MB (ng/mL)	114.1 (52.1, 201.0)	112.8 (51.4, 199.2)	216.9 (165.2, 334.0)	<0.001
CK (U/L)	1,113.1 (300.0, 1,807.5)	1,104.0 (293.3, 1,784.8)	2,081.3 (1,333.7, 3,539.8)	<0.001
LDH (U/L)	480 (343, 652)	477 (341, 645)	825 (558, 1,202)	<0.001
WBC (10^9^/L)	9.6 (7.9, 11.4)	9.6 (7.9, 11.4)	11.5 (10.1, 16.4)	<0.001
N%	73.6 (67.9, 79.0)	73.4 (67.8, 78.9)	83 (77.1, 86.4)	<0.001
RBC (10^12^/L)	4.4 (4.1, 4.8)	4.4 (4.1, 4.8)	4.2 (3.8, 4.6)	0.010
HB (g/L)	137 (126, 147)	137 (126, 147)	131 (119, 142)	0.011
HCT (%)	40.2 (37.3, 42.9)	40.2 (37.3, 42.9)	38.2 (35.3, 40.7)	0.008
PLT (10^9^/L)	216 (184, 254)	216 (184, 254)	211 (184, 252)	0.604
CRP (mg/L)	19.4 (8.8, 31.1)	19.4 (8.7, 31.0)	26.1 (14.8, 42.7)	0.002
ALP (U/L)	70 (58, 84)	69 (58, 84)	74 (59, 84)	0.627
ALT (U/L)	42 (27, 66)	41 (27, 65)	101 (64, 149)	<0.001
AST (U/L)	154 (77, 261)	151 (77, 256)	384 (280, 557)	<0.001
RBG (mmol/L)	8.0 (6.7, 10.4)	8.0 (6.7, 10.3)	11.5 (7.5, 14.1)	<0.001
Cr (umol/L)	68 (59, 80)	68 (59, 79)	96 (80, 127)	<0.001
BUN (mmol/L)	5.8 (4.7, 7.1)	5.8 (4.7, 7.0)	9.3 (7.7, 12.8)	<0.001
UA (umol/L)	331 (275, 395)	330 (274, 394)	422 (336, 507)	<0.001
eGFR [mL/(min·1.73m^2^)]	100.2 (78.1, 123.8)	100.6 (78.9, 124.1)	52.9 (38.3, 85.7)	<0.001
K^+^ (mmol/L)	3.9 (3.7, 4.1)	3.9 (3.7, 4.1)	4.3 (4.0, 4.5)	<0.001
NA^+^ (mmol/L)	140 (138, 141)	140 (138, 141)	141 (137, 142)	0.252
CL^−^ (mmol/L)	104 (101, 107)	104 (101, 107)	102 (99, 106)	0.002
UCB (umol/L)	8.7 (6.3, 11.6)	8.7 (6.3, 11.6)	8.3 (6.2, 11.9)	0.542
TP (g/L)	65 (62, 69)	65 (62, 69)	65 (61, 67)	0.312
ALB (g/L)	40 (38, 42)	40 (38, 42)	38 (34, 40)	<0.001
GLO (g/L)	26 (23, 28)	26 (23, 28)	26 (24, 28)	0.294
TCHOL (mmol/L)	4.5 (3.9, 5.1)	4.5 (3.9, 5.1)	4.4 (3.4, 5.4)	0.517
TG (mmol/L)	1.4 (0.9, 2.0)	1.4 (0.9, 2.0)	1.0 (0.6, 1.7)	0.001
LDL-C (mmol/L)	2.9 (2.4, 3.4)	2.9 (2.4, 3.4)	2.7 (2.0, 3.4)	0.188
HDL-C (mmol/L)	1.0 (0.9, 1.2)	1.0 (0.9, 1.2)	1.1 (0.9, 1.3)	0.071
TSH (mIU/L)	1.5 (0.8, 2.9)	1.5 (0.8, 2.9)	3.2 (1.2, 4.5)	<0.001
LAC (mmol/L)	1.7 (1.2, 2.3)	1.7 (1.2, 2.3)	2.8 (1.8, 5.8)	<0.001
Holter monitor				
MHR (bpm)	70 (64, 78)	70 (64, 78)	85 (75, 98)	<0.001
Max HR (bpm)	103 (95, 113)	103 (95, 112)	119 (105, 142)	<0.001
TVB (times)	35 (4, 387)	38 (8, 259)	889 (41, 2,571)	<0.001
TAB (times)	39 (9, 271)	34 (4, 371)	932 (15, 2,054)	<0.001
Max RR (s)	1.4 (1.2, 1.6)	1.4 (1.2, 1.6)	1.3 (1.0, 1.6)	0.094
Killip classification at admission				
Killip I (%)	3,605 (92.1)	3,577 (92.6)	28 (51.9)	<0.001
Killip II (%)	220 (5.6)	215 (5.6)	5 (9.3)	
Killip III (%)	27 (0.7)	21 (0.5)	6 (22.2)	
Killip IV (%)	64 (1.6)	49 (1.3)	15 (27.8)	

BMI, body mass index; PCI, percutaneous coronary intervention; CABG, coronary artery bypass grafting; HR, heart rate; bpm, beats per minute; RR, respiratory rate; SBP, systolic blood pressure; DBP, diastolic blood pressure; MAP, mean arterial pressure; SpO_2_, blood oxygen saturation; D-to-W, door-to-wire; IRA, infarct-related artery; LM, left main artery; LAD, left anterior descending artery; LCX, left circumflex artery; RCA, right coronary artery; TIMI, thrombolysis in myocardial infarction; IABP, intra-aortic balloon pump; TPM, temporary pacemaker; GP, glycoprotein; ACEI, angiotensin-converting enzyme inhibitors; ARB, angiotensin II receptor blockers; ARNI, angiotensin receptor-erucinase inhibitors; β-B β-receptor blockers; LVEF, left ventricular ejection fraction; BNP, B-type natriuretic peptide; cTnI, high-sensitivity cardiac troponin I; MYO, myoglobin; CK-MB, creatine kinase-MB isoenzyme; CK, creatine kinase; LDH, lactate dehydrogenase; WBC, white blood cells; N%, neutrophil percentage; RBC, red blood cells; HB, hemoglobin; HCT, hematocrit; PLT, platelet count; CRP, c-reactive protein; ALP, alkaline phosphatase; ALT, alanine aminotransferase; AST, aspartate aminotransferase; RBG, random blood glucose; Cr, creatinine; BUN, blood urea nitrogen; UA, uric acid; eGFR, estimated glomerular filtration rate; UCB, unconjugated bilirubin; TP, total protein; ALB, albumin; GLO, globulin; TCHOL, total cholesterol; TG, triglyceride; LDL-C, low-density lipoprotein cholesterol; HDL-C, high-density lipoprotein cholesterol; TSH: thyroid-stimulating hormone; LAC, lactate; MHR, mean heart rate; TVB, total ventricular beats; TAB: total atrial beats.

### Univariate analysis

Univariate logistic regression analysis identified the following factors as significantly associated with in-hospital all-cause mortality (all *p* < 0.05): age, female gender, BMI, initial HR, initial systolic blood pressure (SBP), initial diastolic blood pressure (DBP), initial mean arterial pressure (MAP), SpO₂, emergency hemodynamic instability, IRA [left main artery (LM), left anterior descending artery (LAD)], IABP use intraoperative, TPM use intraoperative, β-B use within 24 h of admission, LVEF, BNP, cTnI, MYO, CK-MB, creatine kinase (CK); lactate dehydrogenase (LDH), white blood cells (WBC), neutrophil percentage (N%), RBC, hemoglobin (HB), CRP, ALT, AST, RBG, creatinine (Cr), blood urea nitrogen (BUN), uric acid (UA), eGFR, K^+^, CL^−^, ALB, triglyceride (TG), lactate (LAC), Holter mean heart rate (MHR), Holter Max HR, Holter total ventricular beats (TVB), Holter total atrial beats (TAB), Killip class (at admission) ≧ II. The results are presented in Table 2.

**Table 2:** One-way logistic regression analysis.

Variable	Wald *χ*^2^	*β*	SE	OR	95% CI	*P* value
Age (per 5 years)	36.608	0.409	0.068	1.505	1.318–1.718	<0.001
Women (%)	5.492	0.666	0.284	1.947	1.115–3.400	0.019
BMI (per IQR kg/m^2^)	13.053	−0.589	0.163	0.555	0.403–0.764	<0.001
Initial vital signs						
HR (per 10 bpm)	13.690	0.230	0.062	1.258	1.114–1.421	0.001
SBP (per10 mmHg)	35.214	−0.255	0.043	0.775	0.713–0.843	<0.001
DBP (per10 mmHg)	20.166	−0.333	0.074	0.717	0.620–0.829	<0.001
MAP (per10 mmHg)	30.148	−0.332	0.060	0.718	0.638–0.808	<0.001
SpO_2_ (per 5%)	9.126	−0.14	0.046	0.87	0.795–0.952	0.003
Emergency hemodynamic instability	42.137	1.919	0.296	6.814	3.818–12.164	<0.001
IRA (LM&LAD)	5.470	0.663	0.284	1.941	1.113–3.385	0.019
IRA Pos-PCI TIMI flow grade <3	3.566	1.144	0.606	3.141	0.958–10.302	0.059
IABP use intraoperative	46.381	2.525	0.371	12.493	6.040–25.840	<0.001
TPM use intraoperative	22.410	2.356	0.498	10.549	3.977–27.979	<0.001
β-B use within 24 h of admission	5.488	−0.901	0.385	0.406	0.191–0.863	0.019
LVEF (per 5%)	68.806	−0.577	0.070	0.561	0.490–0.643	<0.001
BNP (per 200 pg/mL)	61.876	0.356	0.045	1.428	1.307–1.560	<0.001
cTnI (per 10 pg/mL)	28.733	0.231	0.043	1.259	1.158–1.370	<0.001
MYO (per 500 ng/mL)	83.246	0.724	0.079	2.062	1.765–2.409	<0.001
CK-MB (per 100 ng/mL)	70.004	0.811	0.097	2.249	1.860–2.719	<0.001
CK (per 1,000 U/L)	24.694	0.385	0.065	1.470	1.293–1.671	<0.001
LDH (per 200 U/L)	82.642	0.480	0.053	1.617	1.458–1.793	<0.001
WBC (per 5 × 10^9^/L)	43.904	1.084	0.164	2.956	2.145–4.074	<0.001
N% (per 5%)	62.291	0.798	0.101	2.222	1.822–2.709	<0.001
RBC (per 0.5 × 10^12^/L)	7.474	−0.336	0.123	0.715	0.562–0.909	0.006
HB (per 10 g/L)	6.810	−0.203	0.078	0.816	0.701–0.951	0.009
HCT (per 5%)	2.914	−0.243	0.143	0.784	0.593–1.037	0.088
CRP (per 10 mg/L)	26.328	0.162	0.032	1.176	1.106–1.252	<0.001
ALT (per 100 U/L)	6.000	0.091	0.037	1.095	1.018–1.178	0.014
AST (per 200 U/L)	19.913	0.334	0.075	1.396	1.206–1.616	<0.001
RBG (per 5 mmol/L)	27.565	0.605	0.115	1.831	1.461–2.295	<0.001
Cr (per 10 umol/L)	48.417	0.175	0.025	1.191	1.134–1.251	<0.001
BUN (per 1 mmol/L)	128.934	0.406	0.036	1.501	1.399–1.609	<0.001
UA (per 100 umol/L)	50.687	0.786	0.110	2.196	1.768–2.726	<0.001
eGFR [per 10 mL/(min·1.73m^2^)]	64.874	−0.424	0.053	0.654	0.590–0.726	<0.001
K^+^ (per 1 mmol/L)	28.597	1.408	0.263	4.086	2.439–6.845	<0.001
CL^−^ (per 3 mmol/L)	8.226	−0.304	0.106	0.738	0.599–0.908	0.004
ALB (per 5 g/L)	26.423	−0.829	0.161	0.436	0.318–0.599	<0.001
TG (per 1 mmol/L)	7.735	−0.495	0.178	0.610	0.430–0.864	0.005
TSH (per 1 mIU/L)	2.897	0.030	0.017	1.030	0.995–1.066	0.089
LAC (per 1 mmol/L)	86.681	0.453	0.049	1.573	1.430–1.730	<0.001
Holter monitor						
MHR (per 10 bpm)	85.551	0.763	0.083	2.146	1.825–2.523	<0.001
Max HR (per 10 bpm)	64.294	0.423	0.053	1.526	1.376–1.692	<0.001
TVB (per 1,000 times)	7.110	0.078	0.029	1.081	1.021–1.145	0.008
TAB (per1,000 times)	17.477	0.102	0.024	1.107	1.056–1.162	<0.001
Killip Class (at admission) ≧II	77.348	2.456	0.279	11.654	6.742–20.145	<0.001

SE, standard error; OR, odds ratio; CI, confidence interval; BMI, body mass index; IQR, interquartile range; HR, heart rate; bpm, beats per minute; SBP, systolic blood pressure; DBP, diastolic blood pressure; MAP, mean arterial pressure; SpO2, blood oxygen saturation; IRA, infarct-related artery; LM, left main coronary artery; LAD, left anterior descending artery; PCI, percutaneous coronary intervention; TIMI, thrombolysis in myocardial infarction; IABP, intra-aortic balloon pump; TPM, temporary pacing; β-B, β-receptor blockers; LVEF, left ventricular ejection fraction; BNP, B-type natriuretic peptide; cTnI, high-sensitivity cardiac troponin I; MYO, myoglobin; CK-MB, creatine kinase-MB isoenzyme; CK, creatine kinase; LDH, lactate dehydrogenase; WBC, white blood cells; N%, neutrophil percentage; RBC, red blood cells; HB, hemoglobin; HCT, hematocrit; CRP, c-reactive protein; ALT, alanine aminotransferase; AST, aspartate aminotransferase; RBG, random blood glucose; Cr, Creatinine; BUN, blood urea nitrogen; UA, uric acid; eGFR, estimated glomerular filtration rate; ALB, albumin; TG, triglycerides; TSH, thyroid-stimulating hormone; LAC, lactate; MHR, mean heart rate; TVB, total ventricular beats; TAB, total atrial beats.

### LASSO regression

**Figure 2:**
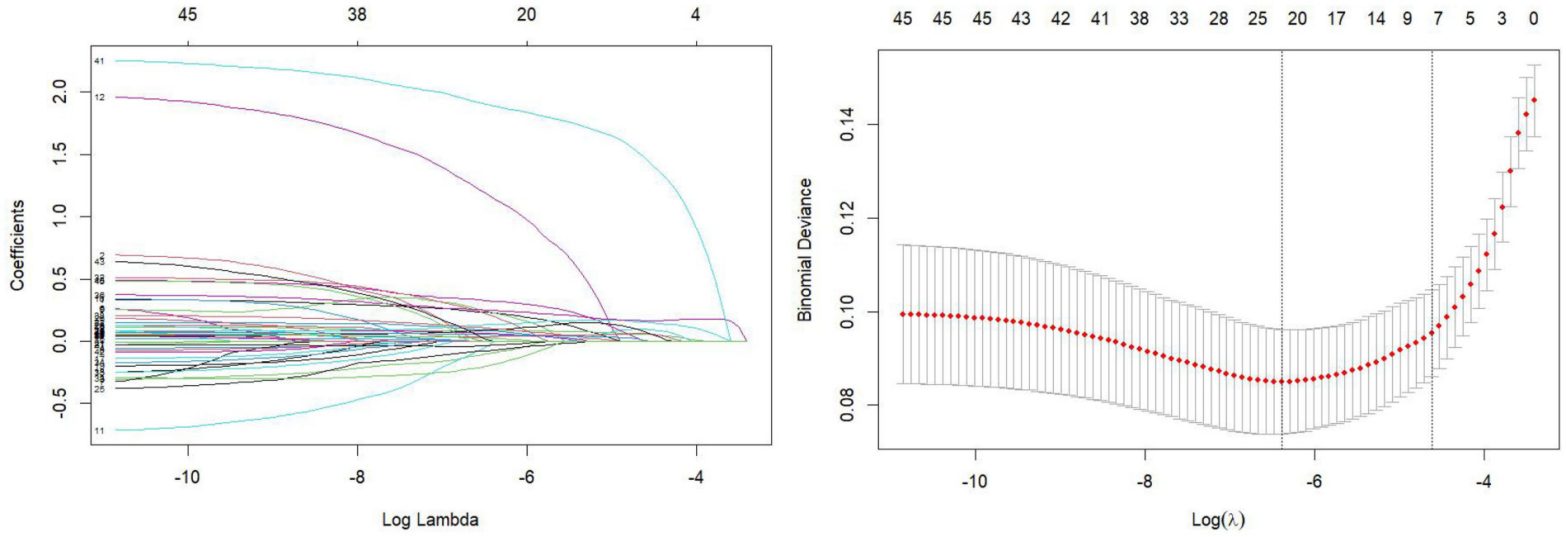
LASSO regression coefficient relationship.

The 42 statistically significant variables were incorporated into LASSO regression analysis, with additional adjustment for history of hypertension, diabetes mellitus, smoking, and alcohol consumption. At the minimum mean squared error (*λ* = 0.002), corresponding to the optimal penalty value indicated by the right vertical dotted line, the analysis identified 9 non-zero coefficient variables: BNP (per200 pg/mL), MYO (per500 ng/mL), CK-MB (per100 ng/mL), LDH (per200 U/L), BUN (per 1 mmol/L), LAC (per 1 mmol/L), Holter MHR (per10 bpm), Holter TAB (per1,000 times) and Killip class (at admission) ≥ II. This model configuration demonstrated optimal fitting performance in the LASSO regression, as illustrated in Figure 2.

### Multicollinearity analysis

Collinearity analysis was performed for these 9 variables, demonstrating tolerance (Tol) values all >0.1 and variance inflation factors (VIF) all <2, indicating no significant multicollinearity among these variables (see Table 3).

**Table 3:** Multicollinearity analysis.

Variable	Tol	VIF
BNP (per 200 pg/mL)	0.862	1.160
MYO (per 500 ng/mL)	0.818	1.222
CK-MB (per 100 ng/mL)	0.639	1.565
LDH (per 200 U/L)	0.632	1.583
BUN (per 1 mmol/L)	0.828	1.208
LAC (per 1 mmol/L)	0.872	1.147
MHR (per 10 bpm)	0.901	1.110
TAB (per 1,000 times)	0.976	1.024
Killip class ≥II	0.886	1.129

Tol, tolerance; VIF, variance inflation factor; BNP, B-type natriuretic peptide; MYO, myoglobin; CK-MB, creatine kinase-MB isoenzyme; LDH, lactate dehydrogenase; BUN, blood urea nitrogen; LAC, lactate; MHR, mean heart rate; bpm, beats per minute; TAB, total atrial beats.

### Multivariate logistics analysis

The nine variables were simultaneously incorporated into a multivariate logistic regression model, which BNP (per 200 pg/mL), CK-MB (per 100 ng/mL), BUN (per 1 mmol/L), LAC (per 1 mmol/L), Holter MHR (per 10 bpm), and Holter TAB (per 1,000 times) as early independent risk factors for in-hospital all-cause death in STEMI patients undergoing primary coronary stenting, with the results visualized in a forest plot and a nomogram. The multivariable logistic regression model for predicting in-hospital mortality can be expressed as:

$$\begin{array}{rl} & \mathrm{Logit}(P)=-8.893+0.145\times \mathrm{BNP}(\mathrm{per}200\;\mathrm{pg}/\mathrm{mL})\\ & \;+0.513\times \mathrm{CK}\text{-}\mathrm{MB}(\mathrm{per}100\;\mathrm{ng}/\mathrm{mL})\\ & \;+0.299\times \mathrm{BUN}(\mathrm{per}\;1\;\mathrm{mmol}/L)\\ & \;+0.230\times \mathrm{LAC}(\mathrm{per}\;1\;\mathrm{mmol}/L)\\ & \;+0.471\times \mathrm{Holter}\;\mathrm{MHR}(\mathrm{per}\;10\;\mathrm{bpm})\\ & \;+0.080\times \mathrm{Holter}\;\mathrm{TAB}(\mathrm{per}1,000\;\mathrm{times}) \end{array}$$

**Figure 3:**
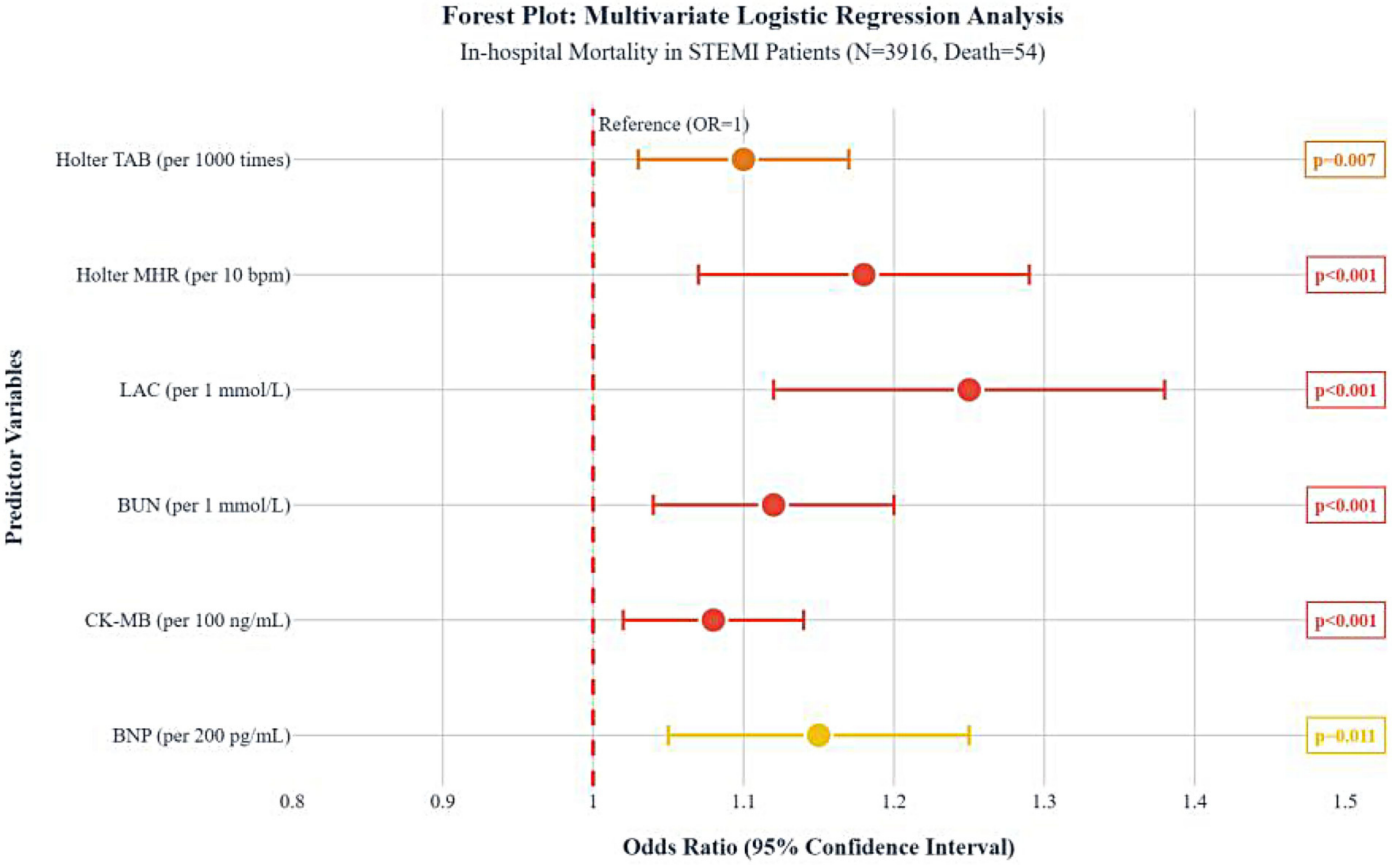
Early prediction model with forest plot for in-hospital death in STEMI patients undergoing primary coronary stenting. STEMI, ST-segment elevation myocardial infarction; OR, odds ratio; BNP, B-type natriuretic peptide; CK-MB, creatine kinase-MB isoenzyme; BUN, blood urea nitrogen; LAC, lactate; MHR, mean heart rate; bpm, beats per minute; TAB, total atrial beats.

**Figure 4:**
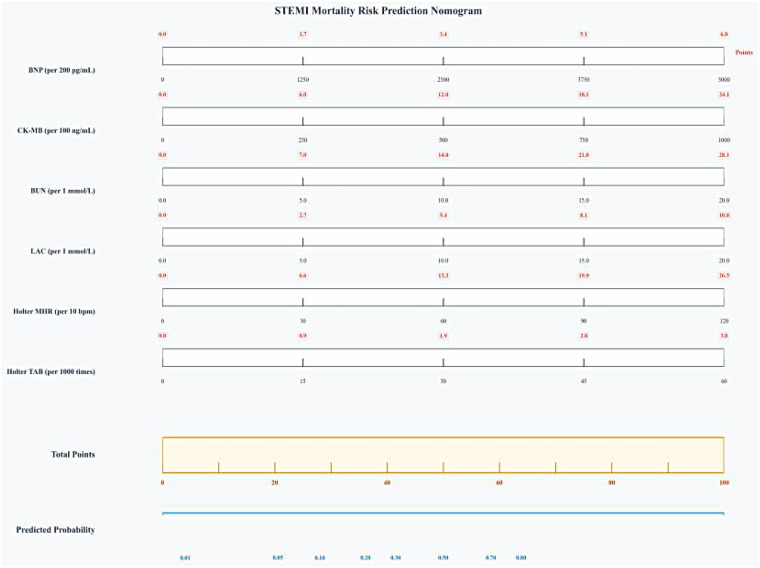
Nomogram for predicting in-hopsital mortality in TEMI patients. STEMI, ST-segment elevation myocardial infarction; OR, odds ratio; CI, confidence interval; BNP, B-type natriuretic peptide; CK-MB, creatine kinase-MB isoenzyme; BUN, blood urea nitrogen; LAC, lactate; MHR, mean heart rate; bpm, beats per minute; TAB, total atrial beats.

The Hosmer–Lemeshow test demonstrated good model fit (*χ*^2^ = 5.610, df = 8, *p* = 0.691), as detailed in Figures 3, 4.

### Predictive efficacy tested by ROC curve

**Figure 5:**
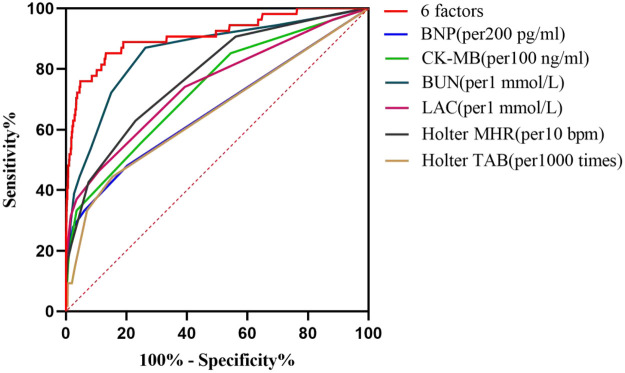
ROC curve analysis of BNP, CK-MB, BUN, LAC, Holter MHR, Holter TAB alone and in combination for predictive modeling. ROC, receiver operating characteristic; BNP, B-type natriuretic peptide; CK-MB, creatine kinase-MB isoenzyme; BUN, blood urea nitrogen; LAC, lactate; MHR, mean heart rate; bpm, beats per minute; TAB, total atrial beats.

ROC analysis demonstrated that the combined model predicted in-hospital all-cause mortality with an AUC of 0.911 (95% CI: 0.863–0.959), significantly outperforming all individual predictors. DeLong's test further confirmed the statistical significance of this superiority: compared to BNP (*D* = 5.956, *p* < 0.001), CK-MB (*D* = 5.031, *p* < 0.001), BUN (*D* = 2.752, *p* = 0.006), LAC (*D* = 4.707, *p* < 0.001), Holter MHR (*D* = 5.040, *p* < 0.001), and Holter TAB (*D* = 5.852, *p* < 0.001). These results consistently indicate that the multi-indicator combination strategy significantly enhances the predictive capability for in-hospital mortality risk in STEMI patients (see Figure 5 and Table 4).

**Table 4:** AUC by ROC analysis.

Variable	AUC	SE	95% CI	Sensitivity (%)	Specificity (%)	*p*
six factors	0.911	0.025	0.863–0.959	0.852	0.868	<0.001
BNP (per 200 pg/mL)	0.664	0.044	0.577–0.751	0.481	0.797	<0.001
CK-MB (per 100 ng/mL)	0.733	0.037	0.660–0.805	0.566	0.752	<0.001
BUN (per 1 mmol/L)	0.855	0.029	0.797–0.912	0.870	0.737	<0.001
LAC (per 1 mmol/L)	0.744	0.039	0.668–0.821	0.463	0.892	<0.001
Holter MHR (per 10 bpm)	0.771	0.033	0.712–0.842	0.630	0.770	<0.001
Holter TAB (per1,000 times)	0.656	0.044	0.571–0.741	0.440	846	<0.001

AUC, area under the curve; ROC, receiver operating characteristic; SE, standard error; CI, confidence interval; BNP, B-type natriuretic peptide; CK-MB, creatine kinase-MB isoenzyme; BUN, blood urea nitrogen; LAC, lactate; MHR, mean heart rate; bpm, beats per minute; TAB, total atrial beats.

### Predictive efficacy tested by DCA

**Figure 6:**
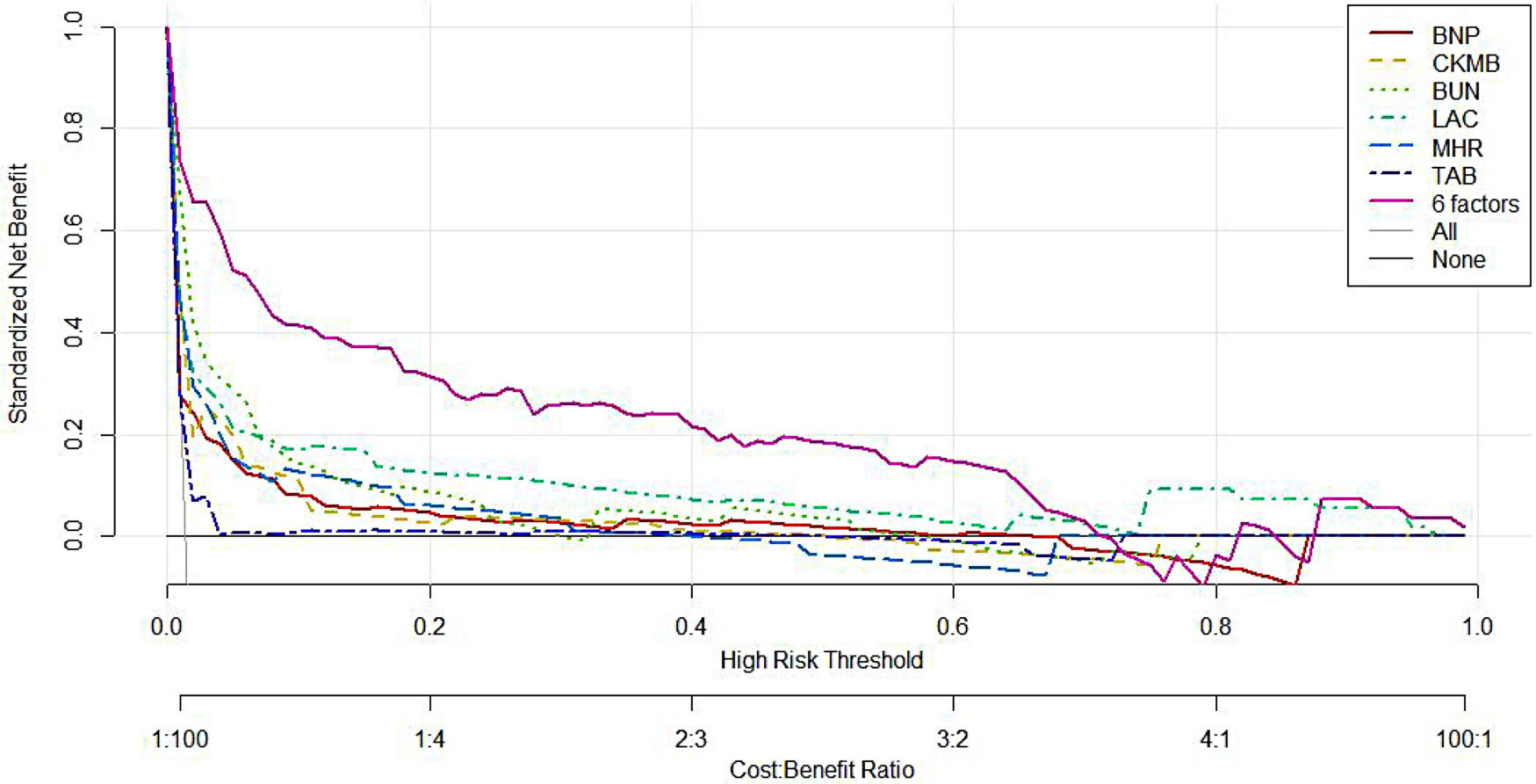
Decision curves of BNP, CK-MB, BUN, LAC, Holter MHR, Holter TAB alone and in combination. STEMI, ST-segment elevation myocardial infarction; BNP, B-natriuretic peptide; CK-MB, creatine kinase-MB isoenzyme; BUN, blood urea nitrogen; LAC, lactate; MHR, mean heart rate; bpm, beats per minute; TAB, total atrial beats.

DCA demonstrated that the purple solid line representing the net benefit of the six-marker combined prediction model consistently remained above both the grey solid line (indicating benefit when all subjects received intervention) and the black horizontal line (representing benefit when no subjects received intervention) across the threshold probability range where it intersected with the two reference lines (purple dashed lines). The model showed superior net benefit compared to any individual biomarker when the threshold probability ranged from approximately 0.010–0.710. This wide probability range indicates substantial clinical utility for the prediction model, as illustrated in Figure 6.

### Internal validation by cross-validation

**Figure 7:**
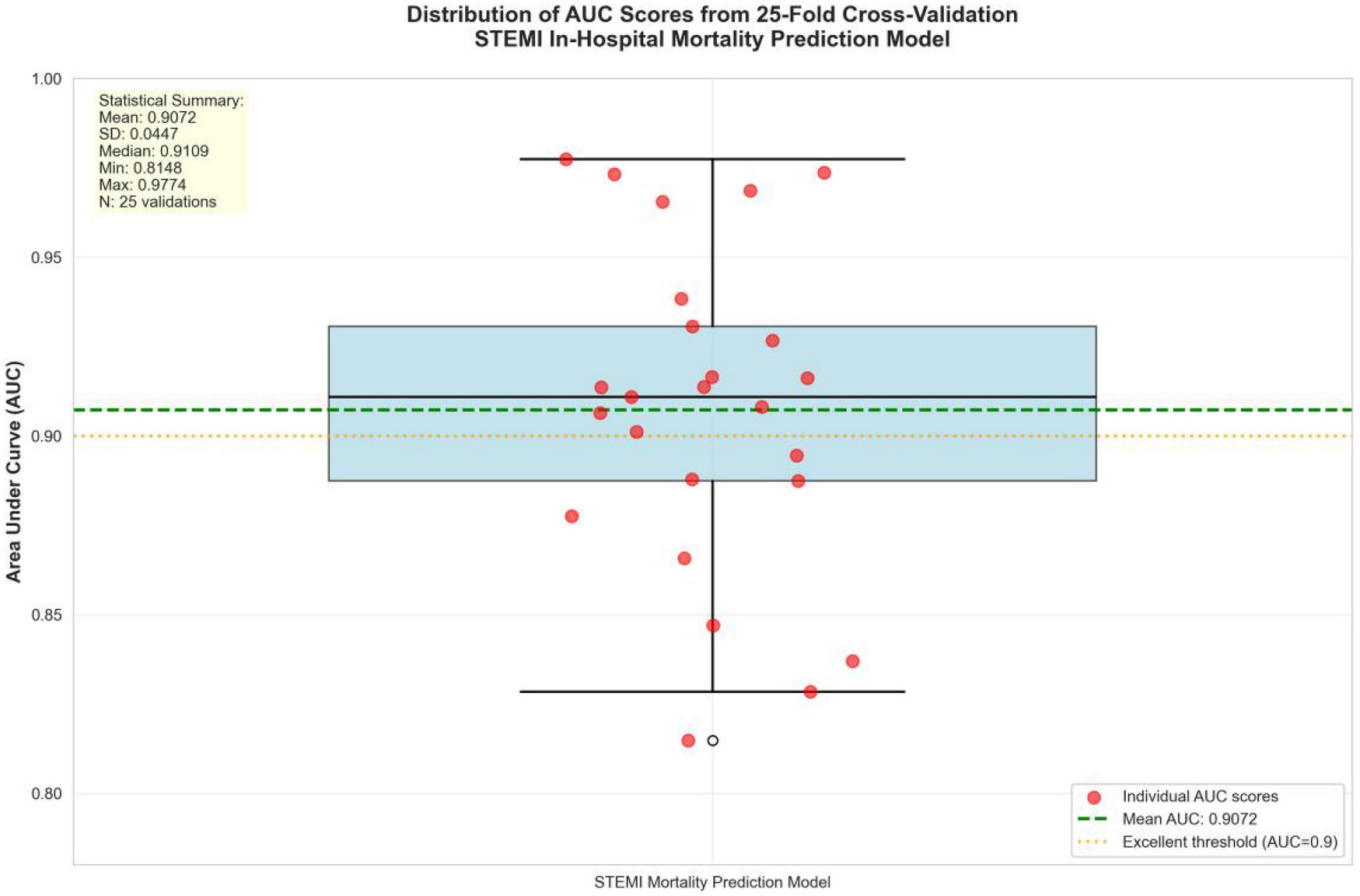
Cross-validation AUC distribution. AUC, area under the curve; SD, standard deviation.

The cross-validation results robustly confirmed the exceptional performance of the prediction model. The mean AUC from 25 validations was 0.911 (standard deviation = 0.045), significantly exceeding the 0.9 threshold for excellent discriminative ability and achieving a maximum AUC of 0.977 in individual validations. The model demonstrated excellent stability, with AUC values consistently distributed in the 0.815–0.977 range (Figure 7). These findings unequivocally demonstrate that the risk assessment model based on six key predictors possesses outstanding discriminatory accuracy and clinical utility, reliably identifying mortality risk in STEMI patients.

### Bootstrap internal validation

**Figure 8:**
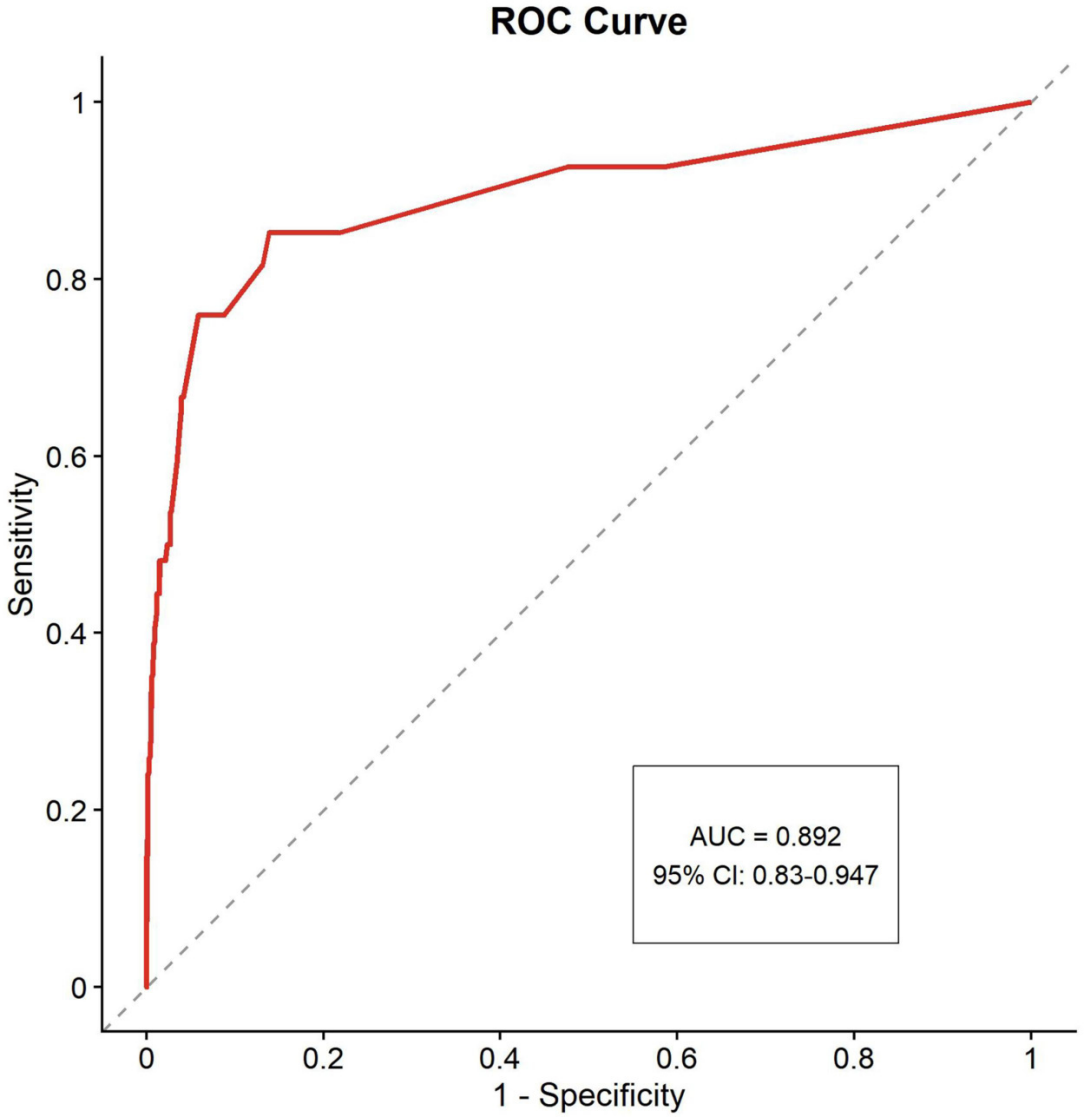
ROC curve with bootstrap optimism correction.

**Figure 9:**
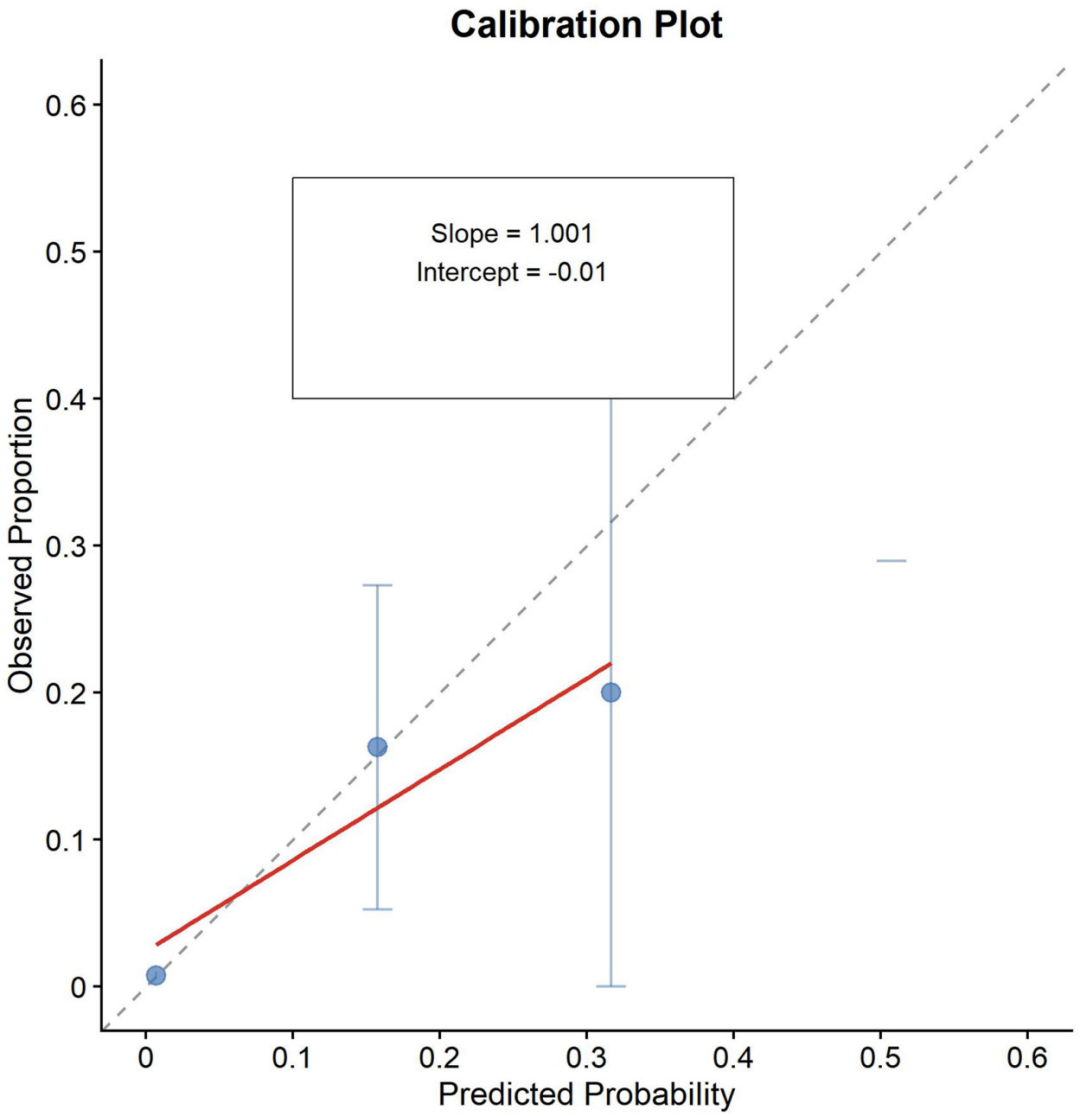
Calibration plot.

To assess potential overfitting given the modest events-per-variable ratio (EPV = 9), we performed bootstrap internal validation with 1,000 repetitions. All 1,000 bootstrap iterations (100%) successfully converged. The bootstrap validation revealed minimal overfitting, with a mean bootstrap AUC of 0.894 (95% CI: 0.830–0.947) and an optimism of 0.019. After optimism correction, the bias-corrected AUC was 0.873 (Figure 8). The bootstrap AUC distribution showed good stability, with a standard deviation of 0.03 and a range from 0.788 to 0.973 (interquartile range: 0.874–0.915). The calibration slope was 1.061, indicating excellent agreement between predicted probabilities and observed outcomes, with values close to the ideal of 1.0 (Figure 9). Despite a borderline EPV of 9, the model can reliably predict in-hospital mortality risk in STEMI patients.

### Simplified model without Holter parameters

To enhance real—world applicability, we developed a simplified prediction model excluding Holter—derived parameters (MHR and TAB). The multivariable logistic regression model retained four independent predictors: BNP (OR = 1.222, 95% CI: 1.095–1.3264, *p* < 0.001), CK-MB (OR = 1.941, 95% CI: 1.563–2.411, *p* < 0.001), BUN (OR = 1.376, 95% CI: 1.262–1.500, *p* < 0.001), and LAC (OR = 1.292, 95% CI: 1.150–1.450, *p* < 0.001) (Supplementary Table S1).

The simplified model achieved an AUC of 0.911 (95% CI: 0.862–0.961) (Supplementary Figure S1, Table S2). Using the Youden index, the optimal cut-off corresponded to a sensitivity of 85.2% and a specificity of 86.8%. The Hosmer–Lemeshow test indicated good calibration (*χ*^2^ = 12.718, df = 8, *p* = 0.122).Decision curve analysis (Supplementary Figure S2) demonstrated that the simplified model still provided a positive net benefit across a clinically relevant range of threshold probabilities (0.001–0.670).

## Discussion

This study analyzed comprehensive clinical data from STEMI patients undergoing primary coronary stenting, identifying elevated levels of BNP, CK-MB, BUN, LAC, Holter MHR, and Holter TAB as independent predictors of in-hospital all-cause death, with the combined predictive performance of these six markers surpassing that of any individual parameter.

Our investigation incorporated 80 clinically relevant parameters for mortality prediction, including general information, medical history, initial vital signs, killip classification at admission, intraoperative data, laboratory tests, bedside echocardiography and 24-h Holter monitor. All these datas (except Holter) were completed within 24 h of hospital admission. The Holter was performed within 48 h. Compared to existing prediction models, this comprehensive early-assessment framework demonstrates superior inclusiveness by systematically incorporating objective clinical parameters available during the critical initial hospitalization period, while maintaining innovative predictive capability through multidimensional data integration.

The bootstrap internal validation confirmed the robustness of our prediction model despite the modest events-per-variable ratio (EPV = 9). The minimal optimism of 0.019 and the narrow confidence interval for the bootstrap AUC (0.830–0.947) indicate that overfitting is negligible and that the model's performance is likely to be preserved in similar populations. The calibration slope of 1.061, very close to the ideal value of 1.0, demonstrates excellent agreement between predicted and observed outcomes, further supporting the model's reliability.

To facilitate clinical implementation, we developed a nomogram (Figure 4) that translates the multivariable logistic regression model into a simple visual tool. In busy clinical settings, clinicians can easily calculate an individual patient's risk score by summing the points corresponding to each predictor. A total score can then be converted to a predicted probability of in-hospital mortality, supporting rapid risk assessment and clinical decision-making. Future development of a web-based calculator or mobile application could further enhance bedside utility.

Compared to the classic GRACE and TIMI risk scores, our novel model developed specifically for STEMI patients undergoing emergency stenting, offers distinct advantages. While GRACE and TIMI are well-validated general tools, their reliance on conventional indicators may limit precision in predicting in-hospital mortality (8, 9). Our model innovatively incorporates dynamic Holter metrics (MHR and TAB) and biomarkers reflecting hemodynamic stress and metabolic state (BNP and LAC). These parameters capture real-time physiological and electrical instability post-intervention, potentially enabling earlier detection of high-risk patients. Thus, our model serves not as a replacement but as a targeted complement to existing scores, enhancing risk stratification for in-hospital death in this specific population.

This study enrolled 3,916 STEMI patients, including 54 in-hospital deaths (incorporating terminal status patients and those discharged automatically), yielding a mortality rate of 1.38%, lower than that reported in most prior studies for STEMI patients undergoing PPCI. PCI procedures comprised two approaches: percutaneous transluminal coronary angioplasty (PTCA) and coronary stenting, with international consensus favoring stenting due to its superior outcomes compared to PTCA alone, which is associated with higher risks of early ischemia/reinfarction, acute target vessel reocclusion, and all-cause mortality (10, 11). However, current guidelines recommend PTCA over stenting in specific clinical scenarios including hemodynamic instability/cardiogenic shock requiring reduced procedure time, high bleeding risk, anatomical constraints (small vessel diameter, diffuse disease, or severe calcification), early post -thrombolysis reperfusion, heavy thrombus burden, in-stent restenosis, or multi-organ failure with limited life expectancy (12, 13). The observed lower mortality in our cohort may be attributed to the exclusive inclusion of stented patients rather than those receiving PTCA alone. Furthermore, as a regional cardiovascular specialty center, our patient population demonstrates clearer disease specificity with fewer complex cases compared to general hospitals, coupled with our interventional cardiologists and coronary care unit (CCU) team's extensive experience, collectively contributing to reduced mortality rates.

BNP, synthesized and secreted by ventricular cardiomyocytes, exerts diuretic, vasodilatory, and anti-fibrotic effects, with its release triggered by cardiac pressure or stretch (e.g., increased ventricular wall tension). Elevated BNP levels demonstrate strong correlations with heart failure and adverse cardiovascular outcomes (14). BUN, a byproduct of protein metabolism, undergoes conversion to urea in the liver via the ornithine cycle before renal glomerular filtration and urinary excretion, with its concentration reflecting the balance between urea production and renal clearance. Previous studies have established that elevated BUN levels significantly correlate with increased risks of major adverse cardiovascular events (MACE) in STEMI patients post-PCI, contributing to prolonged hospitalization and higher mortality (15). LAC, an intermediate glycolytic metabolite, accumulates when severe tissue hypoxia limits oxidative phosphorylation and reduces mitochondrial adenosine triphosphate (ATP) production, leading to pyruvate reduction to lactate by cytoplasmic LDH—thus serving as a byproduct of hypoperfusion or oxygen deprivation. As a key diagnostic marker for cardiogenic shock, elevated LAC positively associates with poor cardiovascular prognosis (16). These established mechanisms align with our findings that elevated BNP, BUN, and LAC levels serve as early predictors of in-hospital all-cause mortality in STEMI patients.

CK-MB, the myocardial-specific isoenzyme of creatine kinase composed of M and B subunits, is predominantly located in cardiomyocytes (accounting for 15%–25% of total cardiac CK) with minimal skeletal muscle presence (<5%). Although traditionally used as a myocardial injury biomarker, CK-MB has been largely superseded by troponin in AMI diagnosis due to inferior specificity and sensitivity. However, beyond diagnostic utility, CK-MB remains valuable for predicting left ventricular remodeling and mortality in AMI patients, with peak levels strongly correlating with infarct size, wall motion abnormalities, left ventricular end-systolic volume index, and fatal outcomes (17). Emerging evidence suggests CK-MB may outperform troponin for prognostic stratification, as demonstrated by a 2023 study showing CK-MB's superior quantitative value for outcome prediction in STEMI patients (18). Our univariate logistic analysis identified both cTnI and CK-MB as significant mortality predictors, but multivariate modeling retained only CK-MB (per 100 ng/mL increase elevating in-hospital death risk by 1.5-fold) as having independent prognostic value.

HR, as a fundamental physiological parameter, demonstrates well-established associations between tachycardia and increased mortality in both healthy individuals and cardiovascular patients (coronary artery disease, hypertension, heart failure). In AMI, elevated HR exacerbate infarct expansion, myocardial ischemia, sympathetic overactivation, cardiac dysfunction, oxygen demand, and atherosclerotic progression—underpinning guideline recommendations for early β-blocker therapy targeting ∼60 bpm resting rates (19). While prior studies predominantly examined random heart rate measurements (admission, in-hospital, discharge), our findings support the clinical utility of Holter-derived parameters in risk stratification of Acute coronary syndrome patients, Consistent with Wang et al. (20) and Duan et al. (21).

Post-revascularization atrial arrhythmias [premature contractions, tachycardia, atrial fibrillation (AF), flutter] frequently complicate STEMI, with >20% of AMI patients having AF history and 5% developing new-onset AF—the latter associated with markedly worse outcomes after PPCI (22). Our study quantified this risk precisely, demonstrating that each 1,000-time increment in 24-h atrial ectopy increased in-hospital mortality by 8.4% (*p* = 0.006), establishing Holter-detected atrial arrhythmia burden as a statistically significant mortality predictor. Therefore, for atrial arrhythmias, maybe ablation improves quality of life and reduces adverse outcomes compared to medical therapy alone. Moreover, multiple studies have confirmed that electrocardiography can predict the prognosis of patients with acute coronary syndromes and heart failure (23, 24). Therefore, incorporating telemonitoring platforms and advanced electrocardiogram analytics could enhance the predictive capacity of our model and support proactive interventions in patients with STEMI.

### Potential limitations

This single-center study has several limitations. First, despite a large overall cohort (*N* = 3,916), the limited number of outcome events (*n* = 54) resulted in an events-per-variable ratio of 9, which is below the conventional minimum threshold of 10. Although bootstrap internal validation suggested acceptable overfitting characteristics (optimism = 0.019, optimism-corrected AUC = 0.873; calibration slope = 1.061), the low EPV remains a concern for potential model instability and overfitting. Therefore, our model should be viewed as exploratory, and independent validation in larger cohorts with more events is essential. Second, the low event rate led to a low positive predictive value (7.9%), limiting the model's ability to accurately identify high-risk patients. Third, due to sparse distributions, some categorical predictors were combined into clinically meaningful categories, which may have resulted in information loss but was necessary given the limited events. Fourth, the study population was restricted to STEMI patients undergoing emergency DES implantation, excluding those receiving PTCA alone, potentially limiting generalizability. Fifth, the lack of post-discharge follow-up data precluded assessment of long-term outcomes. Sixth, this study lacked a direct quantitative comparison with established risk scores (GRACE/TIMI). Without head-to-head assessments, we cannot conclude whether our model provides incremental value over these existing tools. Future studies should include such comparisons. Finally, external validation in independent cohorts is essential before clinical application. Future studies with larger samples, more events, and longer follow-up are warranted to further validate the model.

## Conclusion

Our findings demonstrate that elevated levels of BNP, CK-MB, BUN, LAC, Holter MHR, and Holter TAB are independent predictors of in-hospital all-cause death in STEMI patients. The combined use of these six biomarkers may improve early mortality risk prediction and facilitate timely risk stratification. However, given the absence of external validation, the model's generalizability and clinical utility remain to be established. Further validation in independent multicenter cohorts is warranted before considering clinical implementation.

## Data Availability

The raw data supporting the conclusions of this article will be made available by the authors, without undue reservation.
