# Supplementary material for: Early prediction model for in-hospital all-cause death in patients with acute ST-elevation myocardial infarction after primary coronary artery stenting
Source: Front Cardiovasc Med. 2026 May 19;13:1748053. doi: 10.3389/fcvm.2026.1748053 (PMC13226153; doi:10.3389/fcvm.2026.1748053)
Supplement: Supplementary file 1 [file Table1.docx]

| **Table S1 Multivariate regression analysis of Logistics** | | | | | | |
| --- | --- | --- | --- | --- | --- | --- |
| Variable | *Wald* χ^2^ | *β* | *SE* | *OR* | *95%*CI | *p* |
| BNP (per 200 pg/ml) | 12.847 | 0.201 | 0.056 | 1.222 | 1.095-1.563 | <0.001 |
| CK-MB (per 100 ng/ml) | 36.031 | 0.663 | 0.111 | 1.941 | 1.563-2.411 | <0.001 |
| BUN (per 1 mmol/L) | 52.690 | 0.319 | 0.044 | 1.376 | 1.262-1.500 | <0.001 |
| LAC (per 1 mmol/L) | 18.745 | 0.256 | 0.059 | 1.292 | 1.150-1.450 | <0.001 |
| Note: SE, standard error; OR, odds ratio; CI, confidence interval; BNP, B-natriuretic peptide; CK-MB, creatine kinase-MB isoenzyme; BUN, blood urea nitrogen; LAC, lactate; | | | | | | |


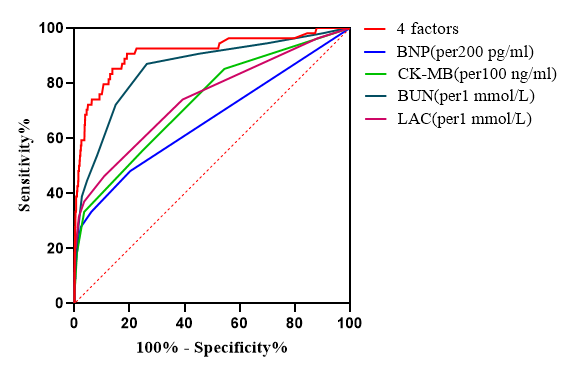


**Fig. S1. ROC curve analysis of BNP, CK-MB, BUN, LAC alone and in combination for predictive modeling.** ROC, receiver operating characteristic; BNP, B-natriuretic peptide; CK-MB, creatine kinase-MB isoenzyme; BUN, blood urea nitrogen; LAC, lactate.

| **Table S2** AUC by ROC analysis | | | | | | |
| --- | --- | --- | --- | --- | --- | --- |
| Variable | AUC | SE | 95% CI | Sensitivity (%) | Specificity (%) | *p* |
| Four factors | 0.911 | 0.025 | 0.862-0.961 | 0.852 | 0.868 | <0.001 |
| BNP (per 200 pg/ml) | 0.664 | 0.044 | 0.577-0.751 | 0.481 | 0.797 | <0.001 |
| CK-MB (per 100 ng/ml) | 0.733 | 0.037 | 0.660-0.805 | 0.566 | 0.752 | <0.001 |
| BUN (per 1 mmol/L) | 0.855 | 0.029 | 0.797-0.912 | 0.870 | 0.737 | <0.001 |
| LAC (per 1 mmol/L) | 0.744 | 0.039 | 0.668-0.821 | 0.463 | 0.892 | <0.001 |
| AUC, area under the curve; ROC, receiver operating characteristic; SE, standard error; CI, confidence interval; BNP, B-type natriuretic peptide; CK-MB, creatine kinase-MB isoenzyme; BUN, blood urea nitrogen; LAC, lactate. | | | | | | |


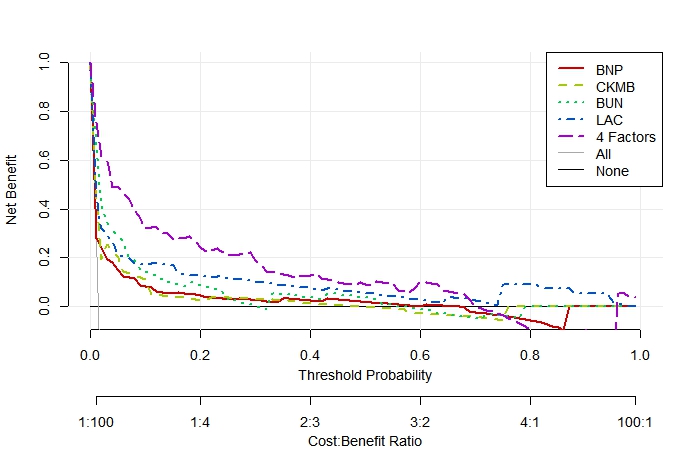


**Fig. S2** Decision curves of BNP, CK-MB, BUN, LAC alone and in combination. STEMI, ST-segment elevation myocardial infarction; BNP, B-natriuretic peptide; CK-MB, creatine kinase-MB isoenzyme; BUN, blood urea nitrogen; LAC, lactate
